# Hamstring Muscle Fatigue and Central Motor Output during a Simulated Soccer Match

**DOI:** 10.1371/journal.pone.0102753

**Published:** 2014-07-21

**Authors:** Paul W. M. Marshall, Ric Lovell, Gitte K. Jeppesen, Kristoffer Andersen, Jason C. Siegler

**Affiliations:** 1 School of Science and Health, University of Western Sydney, Sydney, Australia; 2 School of Medicine and Health, Aalborg University, Aalborg, Denmark; The University of Queensland, Australia

## Abstract

**Purpose:**

To examine changes in hamstring muscle fatigue and central motor output during a 90-minute simulated soccer match, and the concomitant changes in hamstring maximal torque and rate of torque development.

**Method:**

Eight amateur male soccer players performed a 90-minute simulated soccer match, with measures performed at the start of and every 15-minutes during each half. Maximal torque (Nm) and rate of torque development (RTD; Nm.s^–1^) were calculated from maximal isometric knee flexor contractions performed at 10° of flexion. Hamstring peripheral fatigue was assessed from changes in the size and shape of the resting twitch (RT). Hamstring central motor output was quantified from voluntary activation (%) and normalized biceps femoris (BF) and medial hamstrings (MH) electromyographic amplitudes (EMG/M).

**Results:**

Maximal torque was reduced at 45-minutes by 7.6±9.4% (p<0.05). RTD in time intervals of 0–25, 0–50, and 0–75 ms post-contraction onset were reduced after 15-minutes in the first-half between 29.6 to 46.2% (p<0.05), and were further reduced at the end of the second-half (p<0.05). Maximal EMG/M was reduced for biceps femoris only concomitant to the time-course of reductions in maximal torque (p = 0.007). The rate of EMG rise for BF and MH was reduced in early time periods (0–75 ms) post-contraction onset (p<0.05). No changes were observed for the size and shape of the RT, indicating no hamstring peripheral fatigue.

**Conclusion:**

Centrally mediated reductions in maximal torque and rate of torque development provide insight into factors that may explain hamstring injury risk during soccer. Of particular interest were early reductions during the first-half of hamstring rate of torque development, and the decline in maximal EMG/M of biceps femoris in the latter stages of the half. These are important findings that may help explain why the hamstrings are particularly vulnerable to strain injury during soccer.

## Introduction

Examining mechanisms that contribute to muscular fatigue can provide insight into limitations to human sport and exercise performance, as well as the potential causation of musculoskeletal injuries in which fatigue is cited as a primary contributory factor [1]–[3]. Muscular fatigue is often identified as a risk factor linked to the high incidence of hamstring strain injuries observed across all levels of soccer [4]–[6], with injury typically observed in the latter stages of training and each half of competitive matches [1]–[3]. Studies have reported reductions in knee flexor maximal torque during and after actual and simulated soccer match play [7]–[10], with reductions most pronounced in the latter stages of each half [8], [10]. No study has examined whether hamstring fatigue during soccer is mediated by peripheral or central mechanisms.

Mechanisms of fatigue are generally considered from peripheral factors associated with maintaining muscle contraction (e.g. blood flow, oxygen delivery, contraction efficiency), and factors associated with maintaining central motor output to the muscle from the nervous system (e.g. cortical and motoneuron output) [11], [12]. Two studies have examined peripheral muscle fatigue and central motor output 30 to 40 minutes following soccer match play [13], [14]. Both studies reported peripheral muscle fatigue, measured from reductions in the resting twitch elicited from stimulation of the quadriceps [14] and calf muscles [13] respectively, and reduced central motor output measured from declines in voluntary activation. However, neither study performed measurements within the time-course of the match to examine the interaction between peripheral muscle fatigue and central motor output.

Recent evidence from prolonged locomotor activity (e.g. cycling) suggests that increased peripheral feedback into the nervous system (e.g. group III/IV afferent feedback) provides an inhibitory stimulus that reduces central motor output to the muscle during exercise in order to restrict peripheral muscle fatigue and maintain performance [15], [16]. Reduced central motor output to the hamstrings during soccer match play in order to offset peripheral muscle fatigue may explain reductions in knee flexor torque at the end of each half. However, an important consequence of any reductions in central motor output to the hamstrings is the likelihood of concomitant reductions in the ability to rapidly generate knee flexor torque [17].

Reductions in rate of torque development may be important for understanding hamstring injury risk during soccer, particularly at the end of each half. Knee angular velocities during sprinting have been estimated as high as 600 to 700°.s^−1^, thus necessitating rapid hamstring torque development to decelerate knee extension during the terminal swing phase [18]–[20] where hamstring strain injury risk is greatest [21], [22]. To our knowledge one study has reported reduced hamstring rate of torque development following soccer match play with concomitant reductions in the hamstring electromyogram as an estimate of central motor output [9]. However this study [9] did not use stimulation based methods to allow examination of twitch contractile properties as a measure of peripheral muscle fatigue that may also have contributed to declines in hamstring explosive torque [23]. Moreover, this study [9] did not profile the time-course of the change in hamstring rapid torque development during the soccer match. Examining the time-course of changes in rate of torque development during a simulated soccer match may provide greater insight why the hamstrings are vulnerable to fatigue associated injury in the latter stages of each half.

Therefore we designed this study to address critical gaps in the literature pertaining to the understanding of hamstring muscle fatigue and central motor output during soccer and the concomitant changes in hamstring maximal torque and rate of torque development. We used a soccer-specific aerobic field test (SAFT^90^ [8]) to replicate the distances and intensity distribution of elite level players during match play. This provided a controlled experimental context compared to open-field match play where within-game measures are difficult to perform [8], and the within- and between-match locomotor profile is highly variable [24], [25]. We hypothesized that reductions in hamstring central motor output would occur prior to peripheral muscle fatigue during the SAFT^90^. Moreover we hypothesized that reductions in hamstring central motor output would contribute to reductions in both maximal torque and rate of torque development, with the greatest reductions exhibited in the latter stages of each half.

## Materials and Methods

### Subjects

Participants received both verbal and written information concerning the experiment, and all participants gave their written informed consent prior to testing. The study was approved by the University of Western Sydney human research ethics committee, and carried out in accordance with the declaration of Helsinki. Eight healthy male amateur soccer players (age; 22.4 years±4.8, height; 181.2 cm±6.3, body mass; 76 kg±9.9) without any previous hamstring injuries within the last 6 months, participated in the study. The study was carried out during the off-season. On average, the players weekly amount of training during the off-season was 5.1±1.9 h, and included running and strength training.

### General procedures

Participants reported to the temperature controlled laboratory on two occasions where they took part in a familiarization session and one experimental session, which were separated by a minimum of two days. In the 24 h period prior to the testing session participants performed no vigorous exercise or exercise they were unaccustomed to, and refrained from consuming any caffeine or alcohol. Additionally, participants were told to be hydrated upon arrival, and not to consume large quantities of food within 2 h prior to testing. At a preliminary visit to the laboratory, participants were thoroughly familiarized with procedures used to assess knee flexor maximal voluntary isometric torque (isokinetic dynamometer, KinCom 125, Version 5.32, Chattanooga, USA), and peripheral muscle fatigue and central motor output based on the interpolated twitch technique. Participants were also familiarized with the 90-minutes soccer-specific aerobic field test (SAFT^90^), as they completed one 15-minute segment of the protocol [8].

**Figure 1:**
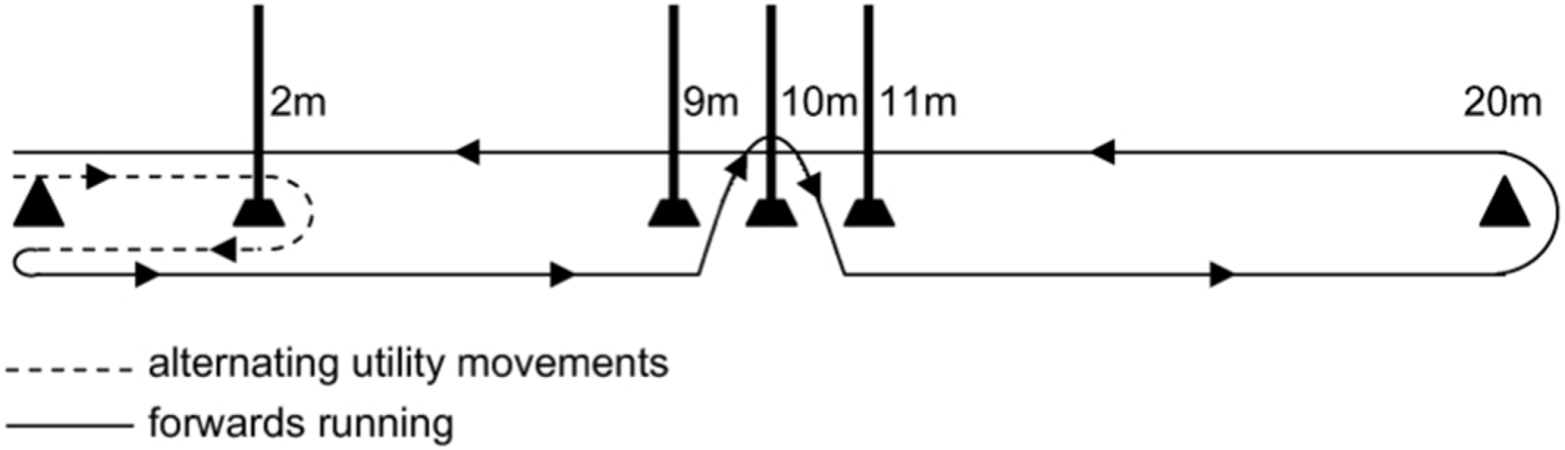
Schematic representation of the SAFT^90^ multidirectional running course.

In the experimental session participants completed the SAFT^90^, during which maximal voluntary torque (MVT) measurements with supramaximal stimulation were obtained prior to SAFT^90^ (T_0_), and every 15-minutes during each half of the protocol (T_15_, T_30_, T_45_ during each half), in addition to a measurement at the end of half-time. The half-time interval was conducted as a passive 15-minute rest and participants remained seated throughout the break. During the experimental session participants were allowed to drink water ad libitum before, at half-time, and after the SAFT^90^. The SAFT^90^ is based on time-motion analysis data from English Championship level match play acquired during the 2007 season [8], and simulates the activity demands and physiological responses of soccer match play [10], [26]. Players navigate around a 20 m agility course (Figure 1) in an intermittent fashion via standing (0 km.h^−1^), walking (∼5.5 km.h^−1^), jogging (∼10.7 km.h^−1^), striding (∼15 km.h^−1^) or sprinting (Maximal effort). The players cover 11.1 km in total, 18.5% of this distance (2.04 km) is performed at high-speed (≥15 km.h^−1^) with 1269 changes in speed (every 4.3 s), and 888 changes in direction (180 degrees) and 444 cutting manoeuvres over the 90 minutes (1332 directional changes). The protocol is divided into equivalent 15-minute activity profiles and was performed on an indoor running surface. The type of movement activity and intensity is controlled using verbal signals from an audio MP3 file [8]. Before commencing the SAFT^90^ participants performed a 15-minute standardized warm-up protocol, which consisted of 12 different soccer specific warm-up exercises. After the warm-up participants rested for 12-minutes before commencing the SAFT^90^, to resemble the pre-match routine of a professional soccer match [27]. Sprint performance was assessed (Swift, Speedlight, Australia) as the average of three maximal 10 m sprints with a 3 m rolling start performed within each 15-minute block of the SAFT^90^.

### Maximal voluntary torque (MVT)

MVT with superimposed supramaximal stimulation was measured at the start of each half, and at 15-minute time intervals during the SAFT^90^. Prior to the start of the first-half, two baseline maximal contractions (T_0_) were performed with 1-minute rest between attempts prior to the 15-minute standardized warm-up protocol. The baseline trial with the highest torque was used for data analysis purposes as the start of the first-half. During all maximal contractions, surface electromyographic (EMG) data were simultaneously recorded.

All voluntary and evoked contractions were performed with the subject lying prone on an adjustable bed. Contractions were performed on the right leg in 10° knee flexion (0° corresponding to full knee extension). The 10° knee flexion angle was selected to provide measurement at an extended muscle length where injury is typically reported to occur during the swing phase of sprinting [21], [22]. The centre of rotation of the dynamometer lever arm was aligned with the femoral condyle of the right knee. The lever arm of the dynamometer was firmly strapped to the lower leg approximately 2 cm superior to the lateral malleolus. Participants were firmly strapped across the hip during contractions to restrict movement. Torque signals were recorded at 2000 Hz using an analog to digital converter (Powerlab 16/35, ADI instruments, Australia; 16-bit analog to digital conversion), and all measurements were corrected for the gravitational effect of the limb. Torque signals were smoothed during offline analysis by a 4^th^ order digital 10 Hz low-pass filter. Each contraction was sustained for 3–4 s. Participants were instructed to flex their knee “as fast and forceful as possible”, and strong verbal encouragement was provided by two investigators during each trial. Prior to baseline measurements (T_0_), participants performed two sub-maximal contractions each at 25, 50 and 75% of self-perceived MVT. During the SAFT^90^ MVT was always measured within 30 s of completing each 15-minute segment.

### Electromyography

Hamstrings electromyograms (EMG) were recorded from the right biceps femoris (BF) and medial hamstrings (MH) using pairs of Ag/AgCl surface electrodes (Maxsensor, Medimax Global, Australia). BF and MH electrodes (10 mm diameter, 10 mm inter-electrode distance) were applied to each muscle after careful skin preparation including removal of excess hair, abrasion with fine sandpaper and cleaning the area with isopropyl alcohol swabs. Placement over BF and MH was in accordance with previous recommendations [28]. The superior electrode was placed longitudinally 35% along a line from the ischial tuberosity to the lateral aspect of the popliteal cavity and 36% along a line from the ischial tuberosity to the medial side of the popliteal cavity for BF and MH respectively. A ground electrode (20 mm contact diameter) was placed on the right fibula head after careful skin preparation. Electrodes were firmly taped to the participant to ensure they remained in place during the SAFT^90^. EMG signals were recorded using the ML138 BioAmp (common mode rejection ratio >85 dB at 50 Hz, input impedance 200 MΩ) with 16-bit analog-to-digital conversion, sampled at 2,000 Hz (ADI instruments, Sydney, Australia). Raw signals were filtered with a fourth-order Bessel filter between 20 and 500 Hz.

### Hamstring muscle stimulation

**Figure 2:**
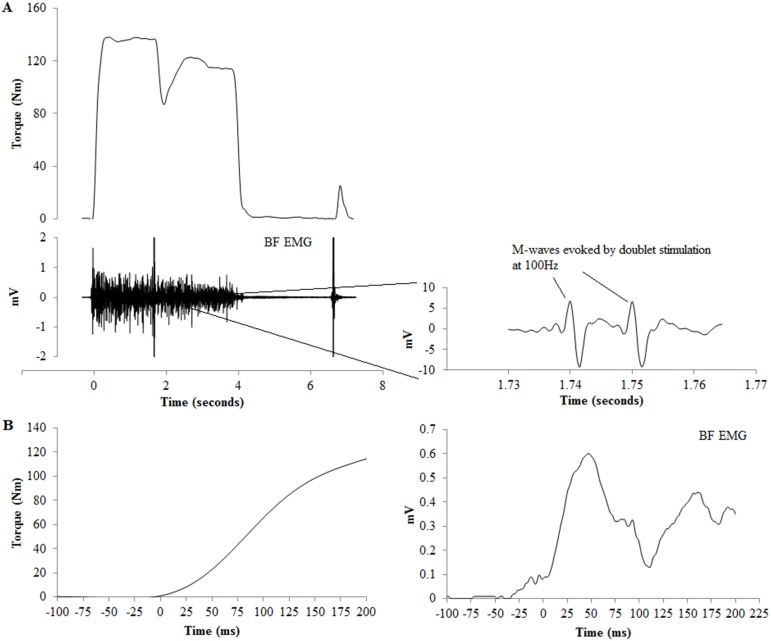
A (top trace), torque and raw electromyography signals recorded during a maximal isometric contraction of the hamstrings. BF, biceps femoris. Time = 0 corresponds to the onset of torque as determined by the integrated profile method. Also displayed are the two evoked M-waves during the maximal contraction, with the peak-to-peak amplitude of the first M-wave calculated for data analysis. B (bottom trace) are the torque and filtered EMG signals (4^th^ order 100 Hz low pass Savitzky-Golay filter) for the same trial as A, in the time period −100 to +200 ms around contraction onset.

Direct muscle stimulation was applied to the hamstrings with surface-stimulating electrodes constructed in the laboratory from aluminum foil, and coated with conduction gel. The width of the stimulating electrodes was 4 cm, and the length (range 11 to 15 cm for cathode, 6 to 8 cm for anode) was sufficient to encase the width of the muscle of both BF and MH. The anode was placed just superior to the popliteal fossa, and the cathode was placed beneath the gluteal fold. Stimulating electrodes were carefully taped down during the SAFT^90^. Stimulation was delivered by a high-voltage stimulator (Digitimer DS7AH, Welwyn Garden City, UK), and consisted of a doublet using two 400-V rectangular pulses (pulse duration 1 ms) with an inter-pulse duration of 10 ms (100 Hz stimulation). During each experimental session, stimulation was initially delivered while participants lay passive and relaxed on the bed, in order to establish the stimulation intensity used during testing. Current was gradually increased in 10 mA increments until a plateau in twitch torque and maximal M-waves (M_max_) for BF and MH were achieved. M-wave amplitude was calculated from the peak-to-peak amplitude of the first evoked M-wave in the EMG signals elicited following stimulation after initial bandpass filtering only (figure 2). This intensity was then used to establish the supramaximal stimulation intensity (130%) that was applied during all maximal contractions in the experimental session (average intensity 176.4 mA±26.9, range; 156–234 mA; between-session reliability for stimulus intensity ICC r-value = 0.98).

For the interpolated twitch technique, one doublet was evoked and superimposed on the maximal contraction when torque had reached a visible plateau. One potentiated doublet was evoked 3–4 s after the maximal contraction was stopped and the participant was relaxed to elicit the resting potentiated twitch (RT). The superimposed twitch (SIT) was calculated as the peak twitch torque in the time period immediately post stimulation subtracted from the average torque 100 ms prior to stimulation. The torque trace resolution was increased to a time epoch 100 ms prior and up to 250 ms post stimulation for careful inspection of a visible evoked twitch as compared to a normal increase in voluntary torque. The SIT was always calculated manually from careful selection and inspection of the respective time periods before and after stimulation. Peak torque of the potentiated twitch was recorded (RT). Voluntary activation (VA) was calculated according to the following equation: VA (%) = [1–(SIT/RT)]×100.

### Data analysis

The onsets of the torque and EMG signals were determined using the integrated profile (IP) method [29]. Onsets were identified within a physiologically relevant window from 75 ms prior to EMG activity (determined visually) and ending 200 ms after EMG onset. The following parameters were identified from each maximal contraction for analysis of the torque signal; 1) the maximal voluntary torque recorded during the contraction prior to stimulation (MVT, Nm), 2) rate of torque development (RTD) was calculated as the average slope of the torque-time curve (Δtorque/Δtime) during the time periods 0–25 ms, 0–50 ms, 0–75 ms and 0–100 ms post contraction onset, 3) maximum RTD was calculated as the greatest average 10 ms slope throughout the contraction.

Prior to EMG data processing signals from BF and MH were digitally smoothed and rectified using a 4^th^ order 100 Hz low pass Savitzky-Golay filter. The following parameters were identified from each MVT trial for BF and MH EMG signals following filtering and onset identification; 1) the rate of EMG rise for BF and MH calculated from the average slope of the EMG-time curve during the time periods 0–25 ms, 0–50 ms, 0–75 ms and 0–100 ms post contraction onset, 2) maximal rate of EMG rise, calculated from the greatest average 10 ms slope throughout the initial 100 ms of the contraction, 3) maximal EMG of BF and MH was calculated as the greatest 250 ms activity throughout the maximal contraction prior to stimulation. All EMG variables were normalized to the respective M-waves elicited during each contraction for data analysis (EMG/M, %).

Using the same onset detection methodology as voluntary torque and EMG analysis, the following parameters were calculated from the resting potentiated twitch; 1) the amplitude of the peak twitch torque, 2) average slope of the torque-time curve during the time periods 0–25 ms, 0–50 ms, 0–75 ms and 0–100 ms post onset of the twitch, 3) maximal twitch RTD based on the greatest average 10 ms slope throughout the twitch, 4) the time to peak twitch, and 5) the half-relaxation time, defined as the time elapsed from the peak twitch torque to 50% peak twitch torque.

### Reliability

From the familiarization session mean within-day, within-subject coefficients of variation for MVT were 2.7±1.9 (range 0.6 to 5.3), VA 1.3±0.7 (range 0.3 to 2.2), and RT 4.9±5.9 (range 0.4 to 14.1). Mean between-day, within-subject coefficients of variation for MVT were 10.4±8.8 (range 0.5 to 25.0), VA 2.4±2.0 (range 0 to 4.5), and RT 7.9±4.7 (range 1.1 to 16.6). No variable was significantly different between testing days. Within- and between-day intra-class correlation coefficients (ICC r-values) were >0.90 for all torque variables including evoked responses, and >0.85 for EMG variables.

### Statistical analyses

All statistical analyses were performed using SPSS statistical software (IBM© SPSS© Statistics, version 22). Data were normally distributed, as assessed by inspection of skewness and kurtosis values and performance of Kolmogorov-Smirnov tests. Repeated-measures analysis of variance procedures (ANOVA) were used to analyze variables over time, with 4 levels of time and 2 levels of half (e.g. T_0_, T_15_, T_30_, T_45_ for each half, where T_0_ for the start of the second half was recorded at the end of half-time). In the event of a significant F ratio, post hoc comparisons were made using a Bonferroni correction. Unless otherwise stated, data are mean ± SD. Two-tailed statistical significance was accepted at p≤0.05.

## Results

### Maximal torque, voluntary rate of torque development, and sprint performance

**Figure 3:**
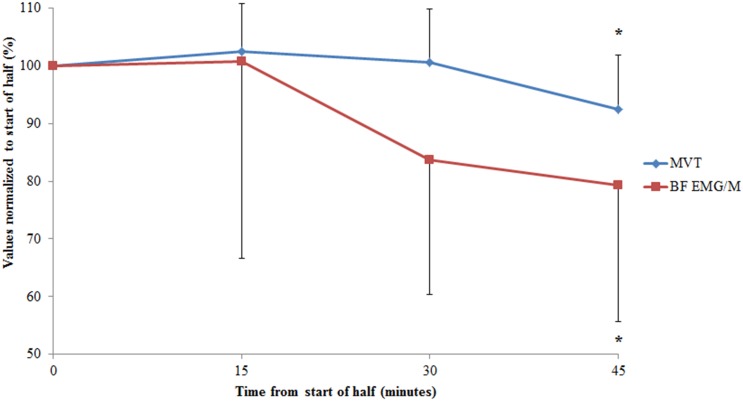
Changes in hamstring maximal voluntary torque (MVT) and biceps femoris (BF) EMG/M values, collapsed across each half at the different time points. Absolute data for each variable (MVT and EMG/M) are normalized to the value recorded before the start of each half. *indicates a significant reduction from the start of the half (p<0.05). Data are mean and SD.

For MVT, no time by half interaction was observed (p = 0.41). A main effect of half was observed with MVT averaged for the first half 15.7 Nm higher than the second half (p = 0.025). A significant time effect was observed (table 1; p<0.001), with MVT at 45-minutes reduced from all other time points (p<0.05; figure 3). The mean reduction at 45-minutes was 7.6%.

**Table 1:** Absolute values for maximal voluntary torque (MVT) and voluntary rate of torque development (RTD) in time intervals post contraction onset throughout the simulated soccer match (SAFT^90^).

		RTD (Nm.s^−1^)				
First-half(minutes)	MVT (Nm)	0–25 ms	0–50 ms	0–75 ms	0–100 ms	RTD Max
0	104.8±18.1	346.7±151.3	469.8±174.9	558.9±168.9	570.1±163.4	790.4±228.3
15	103.8±16.3	177.6±124.5* †	288.8±178.5* †	391.1±218.6* †	450.9±224.2	735.9±288.4
30	101.2±14.4	214.4±108.7	325.9±139.0	427.3±154.0	484.9±152.5	741.9±188.6
45	94.1±9.7*	162.2±116.0*	260.5±155.5*	354.9±187.2*	415.2±179.1	720.1±222.3
**Second-half** **(minutes)**						
0	85.1±12.9	171.3±104.5	239.3±145.1	300.0±171.9	333.3±170.1	535.7±163.0
15	89.1±11.2	188.1±132.4	297.2±164.2	397.7±187.6	452.9±190.9	697.9±235.9
30	87.3±8.0	176.9±107.2	267.1±145.3	353.2±160.7	408.7±142.8	664.3±147.9
45	79.5±13.2*	83.5±70.2*	136.3±98.4*	199.1±121.9*	258.9±132.5	542.4±211.0

*is p<0.05 from the start of the half,

† is p<0.05 for difference between halves for the reduction from the start of the half. Data are mean ± SD.

**Figure 4:**
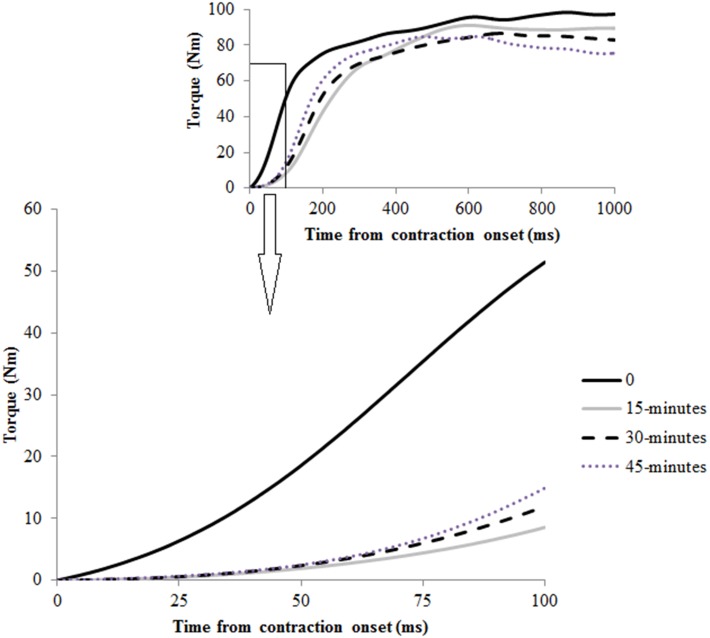
Single subject representation of the typical rate of torque development curves observed during the first-half of the SAFT^90^. Torque data is presented from the point of contraction onset, as defined using the integrated profile method.

Time by half interaction effects were observed for RTD across the time intervals 0–25, 0–50, and 0–75 ms post contraction onset (table 1, figure 4; p<0.05). At 15-minutes in the first half, RTD 0–25, 0–50, and 0–75 ms were reduced from the start of the half by 46.2%, 37.6%, and 29.6% respectively (p<0.05). No reductions from the start of the half were observed at 15-minutes into the second half. At the end of each half RTD 0–25, 0–50, and 0–75 ms were reduced from the start of the half by 29.5%, 21.3%, and 16.0% respectively (table 1, p<0.05). No main effects of time, half, or time by half interactions were observed for RTD 0–100 ms and maximum RTD (table 1).

No main effects of time, half, or time by half interactions were observed for average sprint times within each 15-minute segment of the trial (first 15-minutes, 1.58±0.06 s; last 15-minutes of trial 1.69±0.20 s).

### Central motor output

Voluntary activation at the start of the trial was 97.1±2.2%. No changes were observed for voluntary activation throughout the trial (table 2). A time effect only was observed for BF EMG/M from the maximal torque trials (figure 3; p = 0.007). On average BF EMG/M at 45-minutes was reduced by 20.7% from the start of each half (p = 0.007). In contrast no time, half, or time by half interaction effects were observed for MH EMG/M values (table 2).

**Table 2:** Absolute values for voluntary activation (%), biceps femoris (BF) and medial hamstrings (MH) normalized electromyograms (EMG/M, %), the amplitude of the resting potentiated twitch (RT), time to peak twitch (TPT), and half-relaxation time of the resting twitch (1/2 RT) during the SAFT^90^.

First-half(minutes)	VA (%)	BF EMG/M (%)	MH EMG/M (%)	RT (Nm)	TPT (ms)	½ RT (ms)
0	97.1±2.2	5.2±1.8	4.7±2.1	26.8±5.9	110.1±8.2	63.0±11.2
15	98.0±3.2	4.2±1.5	4.9±2.4	25.3±6.6	104.9±9.2	65.0±19.5
30	98.4±2.6	3.5±0.8	4.3±2.1	25.6±6.1	108.3±11.0	68.4±20.8
45	96.5±3.5	3.6±1.2*	4.4±2.3	25.3±5.9	105.4±10.1	67.6±20.0
**Second-half** **(minutes)**						
0	97.6±3.2	3.4±1.3	4.1±2.0	23.3±5.3	102.8±10.1	65.9±14.1
15	95.7±2.4	3.7±1.3	4.2±1.7	24.6±6.5	104.0±10.6	70.3±21.7
30	96.5±3.4	3.2±1.4	4.2±2.2	24.9±6.6	105.3±13.3	69.4±22.6
45	96.8±2.9	2.9±1.1*	3.9±2.7	24.6±7.6	108.3±14.1	71.5±23.4

*is p<0.05 for the main effect of time for a reduction from the start of the half. Data are mean ± SD.

**Figure 5:**
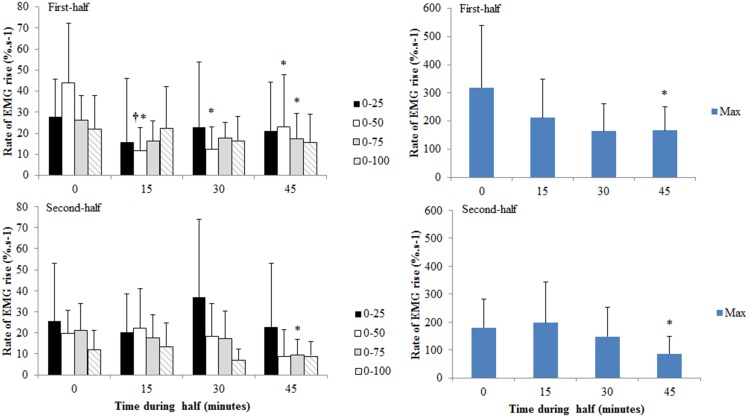
Rate of rise in the biceps femoris EMG signal (EMG/M, %.s^−1^) in time intervals of 0–25, 0–50, 0–75, 0–100 ms, post contraction onset, and maximal rate of rise during the first and second half of the trial. *denotes a significant difference from the start of the half (p<0.05). † is p<0.05 for difference between halves for the reduction from the start of the half. Data are mean and SD.

For BF rate of EMG rise in the time period 0–50 ms, a significant time by half effect was observed (figure 5; p = 0.019). In the first half values were reduced by 66.5% at 15-minutes (p = 0.032), and remained reduced for the rest of the first half. No changes were observed during the second half. A main effect of time was observed for the rate of EMG rise from 0–75 ms (p = 0.009) and maximal BF rate of EMG rise (p = 0.009). The mean reduction at 45-minutes for EMG rate of rise 0–75 ms and the maximal rate were 30.4% (p = 0.048) and 97.9% (p = 0.023) respectively.

**Figure 6:**
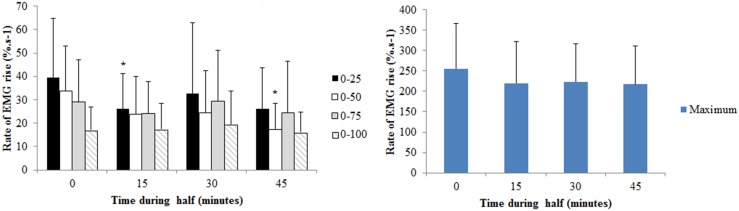
Rate of rise in the medial hamstrings EMG signal (EMG/M, %.s^−1^) in time intervals of 0–25, 0–50, 0–75, 0–100 ms, post contraction onset, and maximal rate of rise at different time points during the half. Data are collapsed across the first and second half. *denotes a significant difference from the start of the half (p<0.05). Data are mean and SD.

Main time effects only were observed for MH EMG rate of rise (figure 6) in time intervals 0–25 ms (p = 0.05) and 0–50 ms (p = 0.027) post-contraction onset. For 0–25 ms, the mean reduction at 15-minutes during each half was 34.3% from the start of the half (p = 0.043). For 0–50 ms, the mean reduction at 45-minutes was 48.6% from the start of the half (p = 0.019).

### Hamstring twitch properties

Maximal M-waves for BF and MH at the start of the trial were 18.1±1.6 mV and 17.0±2.1 mV respectively, and did not change throughout the experiment. The maximal amplitude of the resting potentiated twitch, time to peak twitch, and half-relaxation time of the twitch did not change (table 2). There were no changes observed for the RTD of the potentiated twitch in time intervals of 0–25, 0–50, 0–75, 0–100 ms, and the maximal RTD of the potentiated twitch (table 3).

**Table 3:** Absolute values for rate of torque development (RTD) of the resting potentiated twitch in time intervals post contraction onset throughout the simulated soccer match (SAFT^90^).

	RTD (Nm.s^−1^)				
First-half(minutes)	0–25 ms	0–50 ms	0–75 ms	0–100 ms	RTD Max
0	219.8±67.6	260.0±88.8	278.5±73.6	241.0±60.7	392.8±79.1
15	181.8±98.4	256.1±112.9	274.3±94.6	232.0±65.1	382.5±118.8
30	191.4±99.8	260.1±118.2	276.1±100.9	231.8±62.2	388.4±116.3
45	195.0±89.8	265.4±102.1	280.7±84.5	232.9±56.4	385.3±105.4
**Second-half** **(minutes)**					
0	189.8±87.6	234.6±92.2	244.1±78.6	198.7±58.8	336.5±96.9
15	182.5±96.0	249.9±116.0	263.6±101.1	220.1±65.6	376.1±113.1
30	210.9±89.4	276.1±101.1	281.2±86.2	225.1±65.9	384.0±110.8
45	178.9±91.4	246.4±110.9	262.7±103.7	217.4±79.0	371.6±124.9

No main effects of time, half, or time by half interactions were observed.

## Discussion

### Summary of main findings

We examined changes in hamstring peripheral muscle fatigue and central motor output during a 90-minute simulated soccer match, in addition to changes in maximal torque and rate of torque development. The main results of this study were 1) on average, maximal voluntary torque decreased at the end of the half concomitant to reductions in central motor output to the biceps femoris, 2) no hamstring peripheral muscle fatigue was observed, based on the absence of changes in the size and shape of the resting twitch evoked from the hamstrings, 3) early rate of torque development (0–25, 0–50, 0–75 ms) declined after the first 15-minutes of the first-half concomitant to reduced hamstring muscle central motor output, and thereafter was reduced from the start of each half at 45-minutes.

### Reduced central motor output

For the first time it has been observed that reductions in knee flexor maximal torque and rate of torque development during a simulated soccer match are associated with reduced central motor output to the hamstrings and not peripheral muscle fatigue. The findings of this study conflict with previous reports for reductions in both central motor output and the resting twitch of the quadriceps and plantarflexor muscles 30 to 40-minutes after actual soccer match play [13], [14]. Differences between the studies may, in part, be explained by the use of different muscle groups, and the use of different stimulation methods.

The quadriceps and plantarflexor muscles are prime movers in knee extension and ankle plantarflexion, and thus are involved in many of the explosive movements exhibited in soccer match play (e.g. acceleration, kicking, jumping) and laboratory based simulations (e.g. acceleration, high-speed agility components, change of direction). In comparison, the hamstrings act to decelerate the forward movement of the lower leg during kicking and running motions [18]–[20]. Thus the functional involvement and demands placed on different muscle groups of the lower limb may explain the differences between studies, with the hamstrings lacking the overall contribution to many explosive movements required in the SAFT^90^ that may contribute to peripheral muscle fatigue. Future studies should examine whether similar reductions of the peripheral quadriceps and calf twitch occur following the SAFT^90^ compared to match play findings to validate the demands of the simulation performed in this study, and improve the generalizability of findings reported here.

Differences between studies in the stimulation method applied may also explain why we observed no reduction in the resting twitch. We used direct muscle stimulation to elicit twitch responses from the hamstrings, compared to direct motor nerve stimulation of the femoral [14] and posterior tibial nerves [13]. Comparison of voluntary activation and evoked twitch responses during maximal contractions between different stimulation methods (i.e. direct muscle vs motor nerve) revealed no differences [30], therefore we do not believe the use of direct muscle stimulation explains differences between the studies. A more plausible reason for between study differences in resting twitch changes in the frequency of stimulation used. One study reported reductions in the size and shape of evoked quadriceps twitch responses 40-minutes after soccer match play using single pulses and doublets at 10 Hz, whereas responses elicited with a doublet at 100 Hz similar to this study did not change [14]. While not explicit within the methods, it appears single pulses were used in the recent study that observed reductions in the resting twitch elicited from the calf 30-minutes following match play [13]. In contrast we only used a 100 Hz doublet to elicit twitches based on recommendations for measuring voluntary activation during maximal contractions [31], [32]. The absence of a single pulse or doublet at 10 Hz means we may have profiled the high-frequency component of muscle fatigue, rather than the low-frequency component which has been previously reported following soccer match play [13], [14].

An interesting finding in this study was that reductions in maximal torque at the end of each half occurred concomitant to reduced maximal biceps femoris muscle activity, but not medial hamstrings. This observation may be important because imaging studies of acute hamstring strain injuries in normal and athletic populations report that greater than 80% of injuries are from the biceps femoris [33]–[35]. The biceps femoris, particularly the long-head of the muscle, undergoes the greatest lengthening of all the hamstring muscles during sprinting [36]. Moreover, based on fine-wire EMG findings, the long-head of biceps femoris is more active than all the hamstring muscles at extended knee joint angles [37]. The surface site used in this study probably best represents the activity of the long-head of biceps femoris [37]. Therefore we appear to have observed a muscle specific reduction in central motor output owing to the greater demands placed on the long-head of biceps femoris during the SAFT^90^ at the extended joint angle tested in this study. We are unable to identify the specific mechanism for this finding. While speculative, exercise induced muscle damage following soccer [38], [39] is a stimulant for increased group III and IV afferent feedback [40], [41]. Group III and IV afferent feedback is thought to provide an inhibitory stimulus to central motor output during prolonged locomotor type activity [15], [16]. It may be likely that the combination of greater lengthening during sprinting and higher activity at extended knee joint angles makes the biceps femoris particularly vulnerable to exercise induced muscle damage during soccer. Future studies could examine muscle specific damage after an actual or simulated soccer match to explore the validity of this suggestion.

### Rate of torque development

The results of this study support and extend previous findings for reductions in hamstring rate of torque development during soccer [9]. Reductions in early rate of torque development (0–25, 0–50, 0–75 ms) were observed after only 15-minutes of the first-half. These reductions were maintained through to the end of the second-half, where further decreases were observed. The early first-half reductions in rate of torque development were a surprising finding that do not mirror reported patterns for injury occurrence in the latter stages of each half [1]–[3]. This finding may suggest that reductions in hamstring rate of torque development are not a predisposing factor to hamstring strain injury. Alternatively the early loss of hamstring rapid torque production could be the first mechanistic change that, when combined with the loss of maximal biceps femoris activity and torque output in the latter stage of the half, are the predisposing factors to increased injury risk.

Reductions in rate of torque development were associated with concomitant reductions in central motor output to both hamstring muscles measured in this study. The first-half results for rate of torque decreases were best matched by reductions in early activation of the biceps femoris, which again provides mechanistic insight into why this muscle seems most susceptible to strain injury [33]–[35]. Central motor output is thought to be one of the primary contributory mechanisms to rate of torque development in the early time periods post contraction onset [42]. The results of this study support recent research which suggests that fatigue induced reductions in central motor output have an earlier and more pronounced effect on the ability to rapidly generate torque compared to maximal torque output [17].

The reductions in knee flexor rate of torque development has important implications for the design of sports specific exercise training programs. Current injury prevention and muscle specific conditioning programs in soccer, as well as other team-sports, tend to neglect the development and within-game maintenance of explosive force producing capability. Typically training programs, such as the FIFA-11 [43], [44], concentrate upon the development of eccentric hamstring strength to attenuate the high incidence rate of hamstring muscle strains. The large and early reductions in rate of torque development suggest that intervention strategies to maintain hamstring muscle explosive force generating capacity across soccer match-play should be investigated and applied. To facilitate the design of intervention programs, future research should examine whether hamstring muscle low frequency fatigue occurs. Identification of peripheral contributions to the loss of explosive torque observed in this study could provide a more direct target of training compared to the goal of attenuating declines in early central motor output.

### Methodology considerations

The absence of change in voluntary activation in addition to the observed reduction in normalized EMG/M for biceps femoris may contradict conclusions that central motor output was reduced. However voluntary activation is measured from the evoked torque during the plateau phase of the maximal contraction, and is thus a summation of the additional torque contributions following stimulation of all of the hamstring muscles (short and long-head bicep femoris, semi-membranosus and semi-tendinosus for the medial hamstrings). Thus the measure of voluntary activation should be considered a net estimate of maximal hamstrings central motor output that does not appear to have been compromised during this study.

## Conclusion

Our findings suggest that reductions in knee flexor maximal torque and rate of torque development during a simulated soccer match occurs concomitant to reductions in central motor output to the hamstrings. Of particular interest is that reductions in central motor output manifest as earlier and greater reductions in early rate of torque development compared to reductions in maximal torque. Early rate of torque was reduced after only 15-minutes of the soccer simulation, whereas maximal torque was reduced from the start of each half after 45-minutes. These findings have important implications for understanding injury risk to the hamstrings during soccer.
